# Characteristics of patients who access zero, one or multiple general practices and reasons for their choices: a study in regional Australia

**DOI:** 10.1186/s12875-020-01341-4

**Published:** 2021-01-02

**Authors:** Kristen M. Glenister, John Guymer, Lisa Bourke, David Simmons

**Affiliations:** 1 Research Fellow, University of Melbourne Department of Rural Health. ‘The Chalet’, Docker Street, Wangaratta, 3677 Australia; 2 Wyndham House Clinic, Shepparton, 3630 Australia; 3 University Department of Rural Health, University of Melbourne Department of Rural Health, 49 Graham Street, Shepparton, 3630 Australia; 4 Macarthur Clinical School, Western Sydney University Narellan Road & Gilchrist Drive, Campbelltown, NSW 2560 Australia

**Keywords:** General practice, Continuity of care, Rural, Preventative health, Screening, Lifestyle

## Abstract

**Background:**

Most people in Australia visit a General Practitioner each year and are free to choose their General Practitioner and/or practice on each occasion. A proportion of people visit multiple general practices, which can reduce continuity of care, a core value of general practice. Utilisation of multiple general practices is associated with metropolitan residence and younger age. However, it is unclear which factors are associated with utilisation of multiple general practices in rural areas, where there are often General Practitioner workforce shortages and higher proportions of patients who may benefit from continuity of care, including older people and people living with chronic disease. The aim of this study was to compare the characteristics of people in a rural Australian area who accessed multiple general practices in the previous year with people who had accessed one practice, or none.

**Methods:**

A cross-sectional survey assessed self-reported utilisation and perspective of general practice services, uses of multiple practices, associated reasons, lifestyle advice and screening services received in four regional Victorian towns. Households were randomly selected and residents aged 16+ were eligible to participate in the adult survey.

**Results:**

Most people had attended a single general practice (78.9%), while 14.4% attended more than one practice and 6.7% attended no practices in the previous 12 months. Compared with utilisation of a single general practice, multiple general practice attendance in the previous year was associated with younger age (adjusted odds ratio (aOR 95% confidence interval) 0.98 per year (0.97–0.99), residence in the regional centre aOR 2.90(2.22–3.78), emergency department (ED) attendance in the last 12 months aOR 1.65(1.22–2.21) and no out of pocket costs aOR 1.36(1.04–1.79)). Reasons for multiple general practice attendance included availability of appointments, cost and access to specific services. Compared with multiple general practice attendance, those attending single practices reported more screening tests but similar frequency of lifestyle advice. People who accessed multiple practices were less likely to report very high satisfaction (51.7% vs 62.9% *p* < 0.001) or excellent degree of confidence in their doctor (42.0% vs 49.8% *p* = 0.006) than single practice attendees.

**Conclusions:**

Those attending single practices report higher satisfaction and confidence in their GP and were less likely to attend ED. Further studies are required to test whether increasing availability of appointments and reducing out-of-pocket expenses would increase single practice attendance and/or decrease healthcare costs overall.

**Supplementary Information:**

The online version contains supplementary material available at 10.1186/s12875-020-01341-4.

## Background

The majority of people in Australia (87%) had visited a primary care physician or General Practitioner (GP) at least once during 2016–2017 and on average, people had visited a GP 6.1 times in that time period [1]. People who do not regularly visit a GP may not need to, or may face an access barrier. A systematic assessment of barriers to primary care, reported that availability, after hours services and affordability were particularly important barriers in the Australian context [2]. In Australia, people are free to choose their GP and/or practice on each occasion [3]. Although the majority of people in Australia have a preferred GP [4], a proportion of patients access multiple GPs and multiple practices. An Australian cross-sectional study reported that 11% of adults often visited different GPs [5]. Another Australian cross-sectional study conducted five years later found that 28% of adults had attended more than one general practice in the previous 12 months [6]. Although a study of almost 2500 Australian adults reported that multiple general practice attendance was associated with residence in metropolitan areas, increased risk of attendance at an emergency department (ED) and younger age [6]. Another Australian study of almost 8000 patients reported that patients who visited another practice did so due to appointment availability, convenience of location or for specific health problems [7]. Studies focussed specifically on utilisation of multiple general practices in rural, regional or remote contexts are rare. Utilisation of multiple general practices occurs for a multitude of reasons but may reflect patients exercising choice of healthcare provider, however exercising this choice is complex. A scoping review that assessed determinants of ‘patient choice’ in Western countries reported that younger patients, patients with higher educational attainment or income, or patients with less established relationships with healthcare providers, may be more willing, and/or able, to access different GPs [8]. Some people may be accessing multiple general practices in order to access specific services, such as gynaecological services [9].

Accessing GP care via multiple practices reduces continuity of care. A study of over 230,000 patients in England reported that a high degree of continuity of GP care was associated with reduced hospitalisation for ambulatory care sensitive conditions [10]. Findings from the English GP Patient survey suggest that older people and people with chronic physical or psychological health conditions are more likely to *prefer* continuity of care [11]. Findings from a systematic review of predominantly North American studies suggested that continuity of care was associated with reduced healthcare costs over time, higher levels of patient satisfaction and enhanced preventative medicine [12]. However, continuity of care may be associated with diminished access in the form of reduced availability of appointments [12]. In a systematic review of the barriers to and enablers of achieving continuity of care in rural Australia, continuity of care was reported to be associated with effective communication, availability of resources (including skilled and experienced health care providers) and reduced geographical distance [13].

The Australian GP workforce has become increasingly flexible to accommodate part time work and ongoing training requirements [14] which may impact a patient’s ability to visit the same GP. In rural Australia, continuity of GP care may be even more difficult to achieve due to geographical pockets of GP shortages or high staff turnover [15]. A study of almost 40,000 healthy adults aged 45–74 in Australia suggested that in general, rural people were less likely to receive preventative care, including exercise or dietary advice than their metropolitan counterparts [16] and experience higher prevalence of chronic health conditions compared to people residing in major cities [17] The aim of this study was to estimate the proportion of people in a regional Australian setting who access multiple general practices, their characteristics, their preventative health care, ED presentations and reasons for accessing multiple practices.

## Methods

Crossroads-II [18] was a cross-sectional survey conducted from 2016 to 2018, and studied self-reported health, disease and utilisation of health services among people residing in 4 towns in the Goulburn Valley of regional Victoria, Australia. Households were randomly selected from local government lists. Surveys were conducted face-to-face at the participants’ residence by trained research assistants using RedCap electronic data capture tools (Vanderbilt University) [19]. People were eligible to participate if they had resided in the region for at least six months and were aged 16 years or above. Adults were invited to complete a separate children’s survey for children in their care, but results are not included here. Participating, non-pregnant adults aged 18 years or above were invited to attend a ‘clinic’ at which additional questionnaires were completed. Ethics was granted by the Goulburn Valley Human Ethics Research Committee in May 2016 (GVH20/16). Written consent was obtained from each participant. Participants were asked questions about utilisation and perspectives of GP services, receipt of lifestyle advice and screening tests, ED utilisation and demographic details, as per Additional file 1. Participants were asked ‘If you have visited more than one general *practice* in the past 12 months, please comment why’ and the responses were subsequently coded. A portion of adult participants attended a health screening clinic and were asked additional questions about receiving lifestyle advice and opinion of GP care using several questions from the United Kingdom GP Patient Survey [20] (Additional file 1).

### Analysis

Data were imported into SPSS (SPSS Inc., version 22). Twenty-three participants were excluded from analysis as they did not provide information about the number of general practices visited (*n* = 5) or reported visiting no practices but reported seeing a GP (*n* = 18). Seven participants had visited general practices without seeing a GP and these were included in analysis. Continuous data are presented as mean ± standard deviation, and categorical data as frequencies and percentages. Bonferroni adjustment was used to consider significance for multiple analyses. Independent groups were compared using Student’s t-test. Dichotomous variables were compared using Chi-squared test. The frequency of each code (reason for accessing multiple practices) was assessed within groups of respondents (aged < 65 vs 65+ years, male vs females, smaller towns (populations 6000–9000) vs regional centre (population > 50,000), very satisfied with GP services vs less than very satisfied). Binary logistic regression (direct method) was undertaken to assess the characteristics of people who reported accessing zero vs at least one, or one vs multiple general practices in the previous 12 months (dependent variable). The independent variables were selected if they were significantly different between groups as per Tables 1 and 2. Health status was included as a proxy for general chronic disease status. The independent variables of age, sex, born in Australia, private health insurance, confidence in GPs, attendance at an ED and health status were included in the analysis of attendance at zero vs at least one practice, while the additional independent variables bulk billing (no out of pocket cost to patient), residence in the regional centre or smaller towns, educational attainment (completion of year 12, or less than year 12), frequency of GP visits, GP satisfaction and distance to GP) were included in the analysis of attendance of one or multiple practices.

**Table 1:** Characteristics of respondents who attended zero or at least one general practice in past 12 months

	Did not attend a GP practice in past 12 months		Attended at least one general practice in past 12 months		p
		Missing data (n)		Missing data (n)	
Participants (n, %)	178 (6.7)		2479 (93.3)		–
		0		0	
Male (n, %)	99 (55.6)		1016 (50.4)		< 0.001
		5		7	
Respondents from regional centre (n, %)	86 (48.3)		1249 (49.6)		0.420
		0		0	
Respondents from small towns (n, %)	92 (51.7)		1230 (92.1)		
		0		0	
Age (mean ± SD)	39.5 ± 17.3		54.7 ± 19.0		< 0.001
		4		22	
Completed year 12 or higher (n, %)	108 (63.9)		1313 (57.7)		0.125
		9		205	
Born in Australia (n, %)	139 (78.1)		2087 (84.5)		0.003
		0		9	
Health status (excellent, n, %)	43 (24.3)		308 (12.4)		< 0.001
		1		0	
Bulk billed (n, %)	0		1435 (64.7)		–
		0		262	
Private health insurance (n, %)	73 (41.0)		1148 (46.3)		0.022
		0		0	
Frequency GP visits mean ± SD	0		6.5 ± 8.8		–
		0		269	
Frequency GP visits median, range	0		4.0, 0–112		–
		0		269	
Number of GP visits in previous 12 months:					
• 1 (n, %)			258 (11.7)		–
• 2–3 (n, %)			695 (31.4)		
• 4–11 (n, %)			883 (40.0)		
• 12+ (n, %)			373 (16.9)		
		0		269	
Number different GPs (mean ± SD)	0		2.3 ± 2.6		–
		0		9	
Number different GP practices (mean ± SD)	0		1.2 ± 0.4		–
		0		0	
Very high satisfaction with GP (n, %)	0		1512 (61.2)		–
		0		7	
Excellent level of confidence in GP (n, %)	0		1073 (48.4)		–
		0		264	
Days waiting for appointment (mean ± SD)	0		4.3 ± 8.3		–
		0		81	
Resides less than 5 km from GP (n, %)	115 (80.4)		1952 (83.3)		< 0.001
Resides < 5 km from GP: Regional centre (n, %)	45 (68.2)		911 (77.1)		< 0.001
Resides < 5 km from GP: Smaller towns (n, %)	70 (90.9)		1041 (89.7)		0.038
		35		137	
Attended an ED in past 12 months (n, %)	17 (10.3)		493 (21.0)		0.006
		13		133	

On occasion total equal > 100% due to rounding

Missing data were removed from analysis

**Table 2:** Characteristics of respondents who attended one or multiple general practices in past 12 months

	Attended one GP practice in past 12 months		Attended multiple GP practices in past 12 months		p (1 practice vs > 1 practice)
		Missing data (n)		Missing data (n)	
Participants (n, %)	2096 (78.9)		383 (14.4)		–
		0		0	
Male (n, %)	882 (42.1)		134 (35.0)		0.009
		7		0	
Respondents from regional centre (n, %)	994 (47.4)		255 (66.6)		0.001 (regional centre vs small town)
		0		0	
Respondents from small towns (n, %)	1102 (52.6)		128 (33.4)		
		0		0	
Age (mean ± SD)	56.2 ± 18.7		46.7 ± 18.2		< 0.001
		18		4	
Completed year 12 or higher (n, %)	1077 (56.1)		236 (66.9)		< 0.001
		175		30	
Born in Australia (n, %)	1766 (84.6)		321 (83.8)		0.701
		9		0	
Health status (excellent, n, %)	912 (49.8)		161 (42.0)		0.006
		0		0	
Bulk billed (n, %)	1163 (63.3)		272 (71.4)		0.003
		260		2	
Private health insurance (n, %)	994 (42.3)		154 (40.2)		0.468
		0		0	
Frequency GP visits mean ± SD	6.3 ± 8.6		7.7 ± 9.5		0.007
		267		2	
Frequency GP visits median, range	4.0, 1–112		5.0, 0–99		–
		267		0	
Number of GP visits in previous 12 months:					
• 1 (n, %)	246 (13.4)		12 (3.1)		< 0.001
• 2–3 (n, %)	576 (31.5)		119 (31.2)		0.952
• 4–11 (n, %)	715 (39.1)		168 (44.1)		0.075
• 12+ (n, %)	292 (16.0)		81 (21.3)		0.016
		267		2	
Number different GPs (mean ± SD)	2.2 ± 2.8		2.3 ± 1.1		0.243
		7		2	
Number different GP practices (mean ± SD)	1.0 ± 0.0		2.1 ± 0.3		< 0.001
		0		0	
Very high satisfaction with GP (n, %)	1315 (62.9)		197 (51.7)		< 0.001
		5		2	
Excellent level of confidence in GP (n, %)	912 (49.8)		161 (42.0)		0.006
		264		0	
Days waiting for appointment (mean ± SD)	4.3 ± 8.4		4.5 ± 8.2		0.698
		71		10	
Resides less than 5 km from GP (n, %)	1696 (86.0)		256 (69.2)		< 0.001
Resides < 5 km from GP: Regional centre (n, %)	746 (79.7)		165 (67.1)		< 0.001
Resides < 5 km from GP: Smaller towns (n, %)	950 (91.7)		91 (73.4)		< 0.001
		124		13	
Attended an ED in past 12 months (n, %)	387 (19.6)		106 (28.6)		< 0.001
		120		13	

Missing data were removed from analysis

## Results

Of the 3022 eligible houses (non-vacant, residential properties), at least one response was recorded from 1895 households (63% response rate, 2680 total household participants, data from 2657 participants are included in this study). Of the 1233 adults invited to the ‘clinic’, 748 (61%) attended.

Most participants indicated that they had accessed a GP in the previous 12 months (93%) on an average of 6.5 ± 8.8 occasions (median 4.0, range 0–112). Most participants (*n* = 2096, 78.9%) attended one general practice, while (*n* = 383) 14.4% attended more than one practice and (*n* = 178) 6.7% attended zero practices. When compared with respondents who attended at least one general practice, respondents who reported attending zero general practices were more often male (56% vs 50%, *p* < 0.001), younger (40 ± 17 vs 55 ± 19 years, p < 0.001), of excellent health status (24% vs 12%, p < 0.001) and fewer had attended an ED in the previous 12 months (10% vs 21%, *p* = 0.006), as per Table 1.

Mean waiting time was 4.3 days (median of 2.0, range 0 to 90 days), with no significant difference observed between respondents who accessed single or multiple general practices. Fourteen percent of participants said that they had accessed GPs across multiple practices (average of 2.1 practices, mode = 2, range 2–4) in the previous 12 months. Bulk billing was more commonly reported by users of multiple general practices than single general practices (71% vs 63%, *p* = 0.003). Respondents who utilised single general practices more commonly reported being very satisfied than users of multiple practices (63% vs 52%, *p* < 0.001) and having excellent levels of confidence than users of multiple practices (50% vs 42%, *p* = 0.006). Multiple general practice attendees were more likely to have also presented to ED than attenders of one or zero practice (29, 20, 10% respectively, *p* < 0.005). Respondents from the regional centre were more likely to travel less distance (< 5 km) to the general practice than respondents from the smaller towns, see Table 2.

Patients who utilised one GP practice were more likely to have had their blood pressure, skin, and cholesterol checked in the previous 2 years than patients who utilised multiple practices, as per Table 3. Participants who had not accessed a GP practice in the previous 12 months were significantly less likely to have had screening tests (Table 3) or advice regarding exercise and weight loss (Table 4) than participants who had accessed at least one GP practice. There were very few differences in patient opinion of various aspects of GP care between patients who had accessed zero, one or multiple practices, see Table 4.

**Table 3:** Screening tests reported by respondents attending no practices, one practice or multiple practices in past 2 years (percent) (household participants *n* = 2680)

	Did not attend a GP practice in past 12 months n = 178		Attended a single GP practice n = 2096		Attended multiple GP practices n = 383		p (1 practice vs > 1 practice)	p (0 practice vs ≥1 practice)
		Missing (n)		Missing (n)		Missing (n)		
Blood pressure check	47.2		92.9		90.0		0.046	< 0.001
		2		2		0		
Cholesterol test	22.4		73.7		64.0		< 0.001	< 0.001
		4		0		0		
Diabetes check	22.7		66.9		63.8		0.220	< 0.001
		2		5		0		
Pap test (target group females aged 18–69)	29.2		50.8		58.7		0.247	0.001
		6		97		14		
Bowel check	11.2		41.2		34.9		0.016	< 0.001
		0		3		0		
Bowel check (target group people aged 50–74)	11.5		42.0		36.2		0.189	< 0.001
		0		3		1		
Skin check	16.9		43.6		34.1		< 0.001	< 0.001
		0		0		0		

Missing data were removed from analysis

**Table 4:** Receipt of lifestyle advice and opinion of GP care (clinic participants *n* = 748)

	Did not attend a GP practice in past 12 months *n* = 37		Attended a single GP practice *n* = 605		Attended multiple GP practices *n* = 100		P (1 practice vs > 1 practice	P (o practices vs ≥1 practice)
		Missing (n)		Missing (n)		Missing (n)		
Advice (ever) from GP regarding (%):								
• Exercise	32.4		49.6		45.0		0.410	0.042
		1		32		0		
• Alcohol	5.4		11.0		12.6		0.624	0.414
		2		34		0		
• Diet	35.1		42.1		44.1		0.755	0.394
		0		27		0		
• Weight loss	21.6		39.7		44.5		0.345	0.024
		0		27		1		
• Smoking	8.6		17.3		21.2		0.334	0.175
		2		57		7		
Opinion of GP care (% of participants rating ‘very good’):								
• GP spends enough time	40.6		63.9		60.4		0.501	0.024
		5		79		0		
• GP asks about symptoms	46.7		60.3		50.5		0.078	0.191
		7		84		0		
• GP listens	50.0		65.2		53.5		0.032	0.583
		5		79		0		
• GP explains tests	55.2		62.7		56.8		0.302	0.560
		8		100		6		
• GP involves patient in decisions	45.2		64.3		57.7		0.250	0.058
		6		109		4		
• GP shows care and concern	53.1		67.0		59.6		0.166	0.182
		5		85		2		
• GP takes problems seriously	46.9		66.3		61.4		0.362	0.038
		5		85		0		

Missing data were removed from analysis

The logistic regression model suggested that the significant factors in utilising more than one GP practice were (in order of effect size): age, residence in the regional centre compared with the smaller towns, attendance at an ED, distance to the GP, frequency of GP visits and being bulk billed, see Table 5.

**Table 5:** Likelihood of accessing multiple general practices, compared with a single practice (direct binary logistic regression). Multiple general practices = 1, single general practice = 0

	OR	95% CI	p
Age (per year)	0.981	0.973–0.988	< 0.001
Residence			
Regional centre	2.895	2.218–3.778	< 0.001
Smaller town	1		
Attended ED in past 12 months			
Yes	1.645	1.224–2.211	0.001
No	1		
Distance to visit GP			
≥ 5 km	1		
< 5 km	0.656	0.488–0.882	0.005
Frequency of GP visits in past 12 months (per visit)	1.017	1.003–1.032	0.018
Bulk billed			
Yes	1.361	1.035–1.790	0.027
No	1		
Sex			
• Male	1		
• Female	1.293	0.996–1.678	0.054
Satisfaction with GP			
• Less than very satisfied	1		
• Very satisfied	0.755	0.558–1.022	0.069
Education			
• Completed year 12 or higher	1.252	0.949–1.652	0.112
• Did not complete year 12	1		
Confidence in GP			
• Poor, fair, good or very good	1		
• Excellent	0.787	0.583–1.062	0.117
Health status			
• Poor, fair, good or very good	1		
• Excellent	1.084	0.743–1.581	0.676

This analysis was repeated to assess variables associated with use of no general practices in the previous 12 months, compared with at least one general practice. Attendance at no general practice in the previous 12 months was significantly associated with males (OR 2.09, 1.39–3.16), younger age (OR 0.95, 0.94–0.96), excellent health status (OR 2.39, 1.50–3.80), less confidence in GP (OR 0.56, 0.36–0.86) and less use of ED (OR 0.35, 0.18–0.70), see Table 6.

**Table 6:** Likelihood of accessing zero general practices in past 12 months, compared with at least one GP practice (direct binary logistic regression). Zero practice = 1, at least one practice = 0

	OR	95% CI	p
Sex			
• Female	1		
• Male	2.092	1.385–3.160	< 0.001
Age (per year)	0.952	0.941–0.964	< 0.001
Attended ED in past 12 months			
• vNo	1		
• Yes	0.354	0.179–0.698	0.003
Health status			
• Very good, good, fair or poor	1		
• Excellent	2.387	1.499–3.802	< 0.001
Confidence in GP			
• Very good, good, fair, poor	1		
• Excellent	0.559	0.363–0.861	0.008
Health insurance			
• No	1		
• Yes	0.997	0.644–1.546	0.989
Born in Australia			
• No	1		
• Yes	0.854	0.510–1.431	0.550

The most common reasons for attending multiple general practices (availability of appointments and accessing a subsequent GP for ‘simple’ issues but retaining a preferred GP [5]) were among the top 3 reasons mentioned by each group of respondents. Respondents aged < 65 years more commonly mentioned reasons related to convenience and cost than respondents aged 65+ years. Location of GP services (either convenience or due to patient relocation/travel) were more commonly mentioned by respondents in smaller towns than in the regional centre. Specific services and cost were mentioned more commonly by females than males. Cost appeared to be a factor for respondents who were less satisfied with GP services compared with more satisfied respondents, as per Table 7.

**Table 7:** Most common reasons for attending multiple General Practices according to age, location of residence, sex and satisfaction with GP services. Cells for which less than 10 responses were recorded have been omitted from this table

	Participants < 65	Participants 65+	Smaller towns	Regional centre	Males	Females	Very satisfied	Less satisfied
**1 (most common)**	Availability of appointments (100)	Specific services (16)	Availability of appointments (43)	Availability of appointments (68)	Availability of appointments (40)	Availability of appointments (70)	Availability of appointments (45)	Availability of appointments (66)
**2**	Preferred GP plus subsequent clinic (48)	Preferred GP plus subsequent clinic (14)	Patient moved/was travelling (22)	Preferred GP plus subsequent clinic (41)	Preferred GP plus subsequent clinic (19)	Preferred GP plus subsequent clinic (43)	Preferred GP plus subsequent clinic (30)	Preferred GP plus subsequent clinic (32)
**3**	Convenience (32)	Availability of appointments (11)	Preferred GP plus subsequent clinic (21)	Dr works across multiple sites (29)	Convenience (17)	Specific services (28)	Dr works across multiple sites (25)	Specific services (19)
**4**	Cost (27)	Dr works across multiple sites (12)	Convenience (20)	Specific services (26)	Specific services (15)	Convenience (24)	Specific services (24)	Convenience (19)
**5**	Specific services (26)		Specific services (17)	Cost (21)	Patient moved/was travelling (14)	Dr works across multiple sites (21)	Convenience (22)	Cost (19)
**6**	Dr works across multiple sites (21)		Cost (11)	Convenience (21)	Dr works across multiple sites (13)	Cost (20)	Opening hours (16)	Patient moved/was travelling (16)
**7**	Opening hours (20)			Opening hours (20)	Cost (12)	Patient moved/was travelling (17)	Patient moved/was travelling (15)	
**8**	Patient moved/was travelling (70)					Opening hours (17)	Cost (13)	

## Discussion

We have found that in this rural area, most people had seen a GP in the previous 12 months (93%), slightly higher than the 87% of people reported to had visited a GP at least once during 2015–2016 in Australia [21]. There was evidence that the respondents who had not accessed a general practice in the previous 12 months were more often males, and unsurprisingly, younger people, and/or people reporting excellent health status, likely reflecting episodic GP care for acute health issues. These findings are in agreement with patterns reported in the Australian Bureau of Statistics’ *Patient Experiences* survey [22]. The frequency of visits (6.5 in previous 12 months) was similar to the 6.1 visits per capita reported for 2015–2016 in Australia [23]. The majority of respondents (78%) reported accessing only one general practice, which suggests continuity of informational care (information relevant to the patient’s care is readily available to the patient and healthcare provider). Over half of respondents had seen multiple GPs in this time, reducing relational continuity. The proportion of people attending multiple practices (14%) is similar to the proportion reported by other, albeit predominantly urban studies (11% [4], 19% [21], 28% [5]) and ours is the first study focussed specifically on a regional setting. Participants from the regional centre in this study were significantly more likely to access multiple practices than participants from the surrounding smaller towns. This may relate to a higher number of practices, practices offering a greater variety of services, increased accessibility, bulk billing and choice in the regional centre. In addition, respondents from the regional centre were more likely to live closer to their GP(s) than respondents from smaller towns, particularly for those who accessed multiple general practices.

### Access of multiple general practices

People who attended multiple practices tended to be younger, more likely to be bulk billed, have higher utilisation of ED and reported more frequent GP visits compared with people who attended a single practice. This may point to this group needing to balance a number of competing needs (balancing work or carer commitments, cost considerations), or seeking care from a number of sources. A similar, although predominantly metropolitan, study reported an association between utilisation of multiple general practices and younger people, metropolitan residence and higher education attainment, but no association with bulk billing, and concluded that use of multiple practices was driven by choice rather than cost [5]. Our study suggests that the cost of GP appointments is a factor in a rural setting, perhaps due to the reduced availability of bulk billing compared with metropolitan areas and pockets of socioeconomic disadvantage. This is similar to national data which reports regional areas are more likely to incur out of pocket costs for GP services and were more likely to delay GP services due to cost [24]. GP utilisation by rural men has also been reported to be lower than men in major cities [25].

The exercising of ‘*choice*’ may enable a better fit between the patient and the healthcare provider, but patient choice is complex and reflects a net balance of being willing, and/or able, to choose and actively making a choice of healthcare provider [7]. According to an European based scoping review, patients with higher educational attainment, higher incomes, females, younger age and less established relationships with healthcare providers may be more likely to exercise choice [7]. An Australian study of the characteristics of people utilising multiple general practices identified similar patterns [2]. Utilisation of a subsequent GP practice is likely to be positive for some groups of patients, as their choices are likely to be meeting a specific healthcare need such as a women’s health check with a trusted (often same gender) provider [8], or an appointment for a relatively simple issue that fits around family or work commitments. For other groups of patients such as older people or people with chronic health conditions, care from a single practice appears optimal.

### Reasons for accessing multiple general practices

Reasons for visiting multiple practices primarily related to availability of appointments. In addition, many participants utilised a second practice for specific services such as women’s health checks or for appointments that they considered to be ‘simple’ such as to obtain a medical certificate or prescriptions, in keeping with previous research [26–28].

### Health advice

Rates of self-reported advice regarding common health behaviours were lower than a similar national study [4] but not significantly different between participants who accessed GP services at a single or multiple practices. Advice regarding exercise or weight loss was significantly less common among non-attenders compared with attenders of at least one GP practice.

### ED utilisation

Optimal continuity of care has been reported to be associated with decreased utilisation of EDs [28]. Our results suggest an association between utilisation of multiple general practices and presentation to ED, in agreement with Wright and colleagues [5]. Potentially, this may be due to acute, emergency presentations, injuries, after-hours presentations or need for comprehensive imaging or pathology services [29]. Alternatively, utilisation of ED may be due in part to access barriers to GP services (for example; prohibitive cost of non-bulk billed GP services, extended waiting times or dissatisfaction), or that ED services met a particular need (for example, walk-in service or 24-h care) [29]. Respondents who visited no general practices in the previous 12 months were less likely to present to ED than respondents who attended at least one practice.

### Limitations

This study was conducted in one region in one Australian state, although differences between the regional centre and three smaller towns, each with unique features were assessed. Continuity of GP care is complex, and only limited aspects of practice continuity are explored in this paper. Information provided by participants is likely to be subject to recall bias in relation to utilisation of general practice and ED, and receipt of preventative health care. However, face-to-face data collection is likely to have allowed participation by a wider cross section of respondents than self-administered surveys. A small number of participants reported attending a general practice but not seeing a GP. This may be due to these respondents receiving care from a practice nurse or other health professional. The large sample size increases the generalisability of findings to similar rural areas of Australia.

### Implications

Rural communities in Australia typically have older age structures and higher prevalence of chronic disease than metropolitan communities, and would arguably benefit from high continuity of GP care. However, rural areas of Australia face a multitude of barriers to achieving continuity of care including GP shortages and turnover. Hofer and McDonald have recently outlined practical solutions to increase health service continuity of care in rural Australia including appointment booking procedures that optimise continuity, identification of patients with complex, chronic health conditions, job-sharing and a focus on healthcare staff retention [30]. Although it is difficult to achieve, initiatives that enhance continuity need not come at the expense of prompt appointment access for acute health issues, potentially by leveraging effective triage.

## Conclusions

The results from this study suggest that patients who receive care from a single general practice typically have higher satisfaction and confidence in their GP, are more likely to have screening tests and less likely to go to an ED. Although patients typically have a preferred GP, and following, a preferred practice, a proportion access a GP at another practice, at times due to a lack of appointment availability. Initiatives that enhance continuity of GP care in rural areas may assist to reduce inequities in health outcomes for older people and people living with chronic ill health.

## Supplementary Information

**Additional file 1.**

## Data Availability

The datasets generated and/or analysed during the current study are not publicly available due to ethics approval restrictions but are available from the corresponding author on reasonable request.
