# Supplementary material for: Characteristics of patients who access zero, one or multiple general practices and reasons for their choices: a study in regional Australia
Source: BMC Fam Pract. 2021 Jan 2;22:2. doi: 10.1186/s12875-020-01341-4 (PMC7777414; doi:10.1186/s12875-020-01341-4)
Supplement: Supplementary file 1 — Additional file 1. [file 12875_2020_1341_MOESM1_ESM.docx]

Appendix 1: Questions relevant to this study.

|  | Question | Answers |
| --- | --- | --- |
| 1 | In the past 12 months have you visited a GP- General Practitioner | Yes, No |
| 2 | Which GP clinics have you visited in the past 12 months? | List of 28 local practices, plus ‘other, please specify’, select all that apply. |
| 3 | If you visited more than one GP clinic, please comment why? | Free text |
| 4 | Are you bulk-billed? | Yes, No |
| 5 | In the past 12 months how often did you visit a GP? |  |
| 6 | In the past 12 months how many GPs have you visited? |  |
| 7 | Please comment why you went to different GPs? | Free text |
| 8 | How many days did you wait for an appointment with the GP? (From the time you rang to make the appointment to the data of the appointment, not in the waiting room) |  |
| 9 | In the past 12 months how satisfied were you with the GP? | Very satisfied, satisfied, neither dissatisfied or satisfied, dissatisfied, very dissatisfied. |
| 10 | My confidence in the doctor’s ability: | Excellent, very good, good, fair, poor. |
| 11 | What is your sex? | Male, female, transgender/intersex/other |
| 12 | What is your date of birth? | Age calculated (years) between date of birth and survey date. |
| 12 | Were you born in Australia? | Yes, No |
| 13 | Do you have private health insurance coverage for hospital expenses? | Yes (minimal coverage), yes (higher coverage) recoded as yes. No, Department of Veterans affairs card, health care card, recoded as no. |
| 14 | How far do you generally travel for the following services: (GP) | less than 5km, 5-10km,11-50km,50-100km, >100km, NA |
| 15 | Have you been to the Emergency Department to receive medical care in the past 12 months? | Yes, No |
| 16 | What is the highest level of education that you have achieved? | A list of 17 levels, recoded into less than year 12 and year 12 or higher. |
| 17 | In general would you say that your health is: | excellent, very good, good, fair, poor. |
| 18 | In the past 2 years have you had any of the following check-ups (Blood pressure check, Cholesterol check, Test for diabetes or high blood sugar, Bowel examination, Skin examination (for lesions/cancers), Mammogram (breast x-ray), Pap smear test, Prostate check) | yes, no, don’t know, NA |
| 19 | Has a doctor or other health professional ever advised you to quit smoking? | Yes (within past 12 months), yes (within past 3 years), yes (3 or more years ago), no, don’t know. Recoded into yes, no, don’t know omitted. |
| 20 | Has a doctor or other health professional ever talked to you about physical activity or exercise? | Yes (within past 12 months), yes (within past 3 years), yes (3 or more years ago), no, don’t know. Recoded into yes, no, don’t know omitted. |
| 21 | Has a doctor or other health professional ever talked to you about whether the amount of alcohol you drink was a problem? | Yes (within past 12 months), yes (within past 3 years), yes (3 or more years ago), no, don’t know. Recoded into yes, no, don’t know omitted. |
| 22 | Has a doctor or other health professional ever talked to you about your diet or eating habits? | Yes (within past 12 months), yes (within past 3 years), yes (3 or more years ago), no, don’t know. Recoded into yes, no, don’t know omitted. |
| 23 | Have you ever been told by a doctor or nurse that you should lose weight? | Yes, no, don’t know |
| 24 | Last time you saw a doctor at your GP surgery or health centre, how good was the doctor at each of the following?  (giving you enough time, asking about your symptoms, listening to you, explaining tests and treatments, involving you in decisions about your care, treating you with care and concern, taking your problems seriously) (Campbell et al, 2009). | Very good, good, neither good nor poor, poor, very poor, doesn’t apply |
